# Audio-Visual Spatiotemporal Perceptual Training Enhances the P300 Component in Healthy Older Adults

**DOI:** 10.3389/fpsyg.2018.02537

**Published:** 2018-12-11

**Authors:** Weiping Yang, Ao Guo, Yueying Li, Jiajing Qiu, Shengnan Li, Shufei Yin, Jianxin Chen, Yanna Ren

**Affiliations:** ^1^Department of Psychology, Faculty of Education, Hubei University, Wuhan, sChina; ^2^Brain Cognition Research Center (BCRC), Faculty of Education, Hubei University, Wuhan, China; ^3^Department of Psychology, Medical Humanities College, Guiyang College of Traditional Chinese Medicine, Guiyang, China

**Keywords:** P300, perceptual training, audio-visual stimuli, older adults, audio-visual integration, spatiotemporal

## Abstract

In older adults, cognitive abilities, such as those associated with vision and hearing, generally decrease with age. According to several studies, audio-visual perceptual training can improve perceived competence regarding visual and auditory stimuli, suggesting that perceptual training is effective and beneficial. However, whether audio-visual perceptual training can induce far-transfer effects in other forms of untrained cognitive processing that are not directly trained in older adults remains unclear. In this study, the classic P300 component, a neurophysiological indicator of cognitive processing of a stimulus, was selected as an evaluation index of the training effect. We trained both young and older adults on the ability to judge the temporal and spatial consistency of visual and auditory stimuli. P300 amplitudes were significantly greater in the posttraining session than in the pretraining session in older adults (*P* = 0.001). However, perceptual training had no significant effect (*P* = 0.949) on the P300 component in young adults. Our results illustrate that audio-visual perceptual training can lead to far-transfer effects in healthy older adults. These findings highlight the robust malleability of the aging brain, and further provide evidence to motivate exploration to improve cognitive abilities in older adults.

## Introduction

In a natural environment, perceptual events often provide information from multiple sensory modalities, and the integration of this information is an essential component for cognition. For example, a crashing ball reflects light to our eyes at the moment the ball strikes the ground and creates air-borne vibrations that are transmitted to our ears. Bimodal audio-visual stimuli can be discriminated or detected more accurately and faster than unimodal auditory or visual stimuli (Giard and Peronnet, 1999; Molholm et al., 2002). Behavioral and perceptual benefits are influenced by spatial (locational relationship of visual and auditory stimuli) and temporal (time synchronous relationship between visual and auditory stimuli) factors (Stevenson et al., 2012). In line with this behavioral evidence, electrophysiological studies have revealed that audio-visual integration occurs earlier and is greater in multisensory brain areas when auditory and visual stimuli are presented in spatial and temporal coincidence (Stein and Meredith, 1993; Yang et al., 2013). For older adults, the auditory threshold tends to increase, and visual acuity generally decreases with aging. Based on electrophysiological results, the amplitude of responses to audio-visual stimuli is reduced in older adults, corresponding to a slower response time and reduced reaction facilitation effects (Stephen et al., 2010). Older adults have greater difficulty in discriminating temporal order than do younger adults, leading to a larger temporal binding window (Bedard and Barnett-Cowan, 2015). Although sensory discrimination in addition to attention (Mahoney et al., 2011) and localization functions (Freigang et al., 2015) is attenuated in older adults, whether this cognitive processing can be improved through training remains an open question.

Recently, some researchers investigated whether audio-visual perceptual training paradigms in which participants were given feedback about the correctness of their simultaneity judgments could alter the temporal characteristics of audio-visual processing. Their findings indicated that audio-visual temporal perceptual training narrows the temporal window of multisensory binding, and suggested that multisensory temporal processing has a high degree of flexibility (Powers et al., 2009). Setti et al. (2014) trained older adults to judge the temporal order of visual and auditory stimuli and found that audio-visual temporal discrimination training reduced the susceptibility to a multisensory illusion, with a sound inducing a flash illusion (Setti et al., 2014). According to evidence from electrophysiological and imaging studies, audio-visual multisensory training enhances visual processing of motion stimuli (Grasso et al., 2016). The findings described above, such as the narrowing of the temporal window or enhanced multisensory integration, are plausible explanations to account for bimodal audio-visual training effects. Interestingly, this training effect can be extended to other untrained capabilities, which is called the “far-transfer effect,” suggesting possibilities for rehabilitating optimal audio-visual perceptual function with a positive impact on a number of cognitive abilities, including attention. Additionally, attention plays an important role in audio-visual integration processes, and audiovisual integration under attended conditions is greater than that under unattended conditions (Talsma and Woldorff, 2005; Koelewijn et al., 2010; Talsma et al., 2010). However, whether repetitive audio-visual stimulation would change cognitive faculties, such as attention, remains unclear. Therefore, in the current study, we used auditory P300 to assess cognitive ability. Event-related potentials (ERPs) were recorded, and time-locked segments of electroencephalography (EEG) activity reflected the discrete stages of information processing. P300, an ERP component that provides an index of attentional resources, was evoked using an auditory oddball paradigm.

The classic P300 component is a neurophysiological indicator of the cognitive processing of a stimulus. P300 includes at least two subcomponents, P300a and P300b. P300a is larger frontally and occurs earlier (latency 220–280 ms) than centro-parietal P300b (latency 310–380 ms) (Squires et al., 1975). P300a is often elicited by rare distractor stimuli and represents stimulus-driven frontal attention mechanisms (Polich, 2007). P300b is often driven by target detection paradigms and is associated with controlled processing (Halgren and Marinkovic, 1994). The P300 component is elicited by attended and task-relevant stimuli (Polich, 2007). Thus, the amplitude of P300 served as our covert measure of attention that arises independently of behavioral responses (Gray et al., 2004). In addition, the P300 component is suitable for studying elderly subjects because it is relatively easily elicited by several low-probability events. In this study, the traditional auditory oddball paradigm was used, in which a participant is presented with a sequence of auditory stimuli representing two categories, 1000 and 2000 Hz. Auditory stimuli at 2000 Hz were presented less frequently and were used to evoke the P300 component.

To confirm whether bimodal training affects the P300 component of older adults, we designed an audio-visual spatiotemporal discrimination task that included visual and auditory stimuli presented at different/same locations and times. Before training, the P300 components elicited by an auditory oddball paradigm in both young and older adults were recorded and defined as pretraining. Then, all participants conducted perceptual training for a month. The training was implemented 4 days a week and lasted from 10 to 20 min each day. After training, the auditory oddball test was measured in the two groups again and defined as posttraining. By comparing the P300 component between the pre- and posttraining sessions, we determined whether a decline in cognitive processing could be improved through audio-visual spatiotemporal perceptual training.

## Materials and Methods

### Participants

Fifty-two participants were included in this study. Twenty-six healthy older adults were equally divided into a training group (68–75 years of age, mean age: 70.7 years) and a control group (65–78 years of age, mean age: 68.1 years). Twenty-six young adults were also equally divided into a training group (19–21 years of age, mean age: 20.1 years), and a control group (19–21 years of age, mean age: 20.5 years). The mini-mental state examination (MMSE) and Montreal cognitive assessment (MoCA) were used to evaluate participants’ cognitive functioning. Individuals provided written informed consent prior to participating in the study, which was previously approved by the Ethics Committee of Hubei University.

### Experimental Design

Stimulus presentation and response collection were accomplished using Presentation software (Neurobehavioral Systems Inc., Albany, CA, United States). The streams of two types of auditory stimuli (1000 and 2000 Hz) were randomly presented. High-pitched tones were assigned as target stimuli, and low-pitched tones served as non-target tones. A total of 200 pure tones (10 ms of rise and fall) were presented binaurally via earphones (CX-300, Sennheiser, Japan) at an intensity of 65 dB SPL. Target stimuli were presented at a frequency of 20% of the total stimuli. The duration of each type of stimulus was 50 ms. The interstimulus interval (ISI) varied randomly between 900 and 1100 ms (mean ISI = 1000 ms).

During the experiment, participants were required to open and fix their eyes on a centrally presented fixation point on a 21-inch computer monitor positioned at a viewing distance of 70 cm to avoid alpha rhythm synchronization. The participants’ task was to count the target tones silently while ignoring the non-target tones. After the investigation, participants were asked to report the number of targets. Before the recording session, the task was explained and a practice block was provided to each participant to ensure a good level of performance. The participants underwent testing twice, including a pretest (baseline) and posttest.

### Audio-Visual Perceptual Training

The training was performed in a dimly lit, sound-attenuated room (laboratory room, Hubei University, China). Participants were required to sit at a distance of 70 cm from a monitor with a refresh-rate of 60 Hz. A stimulus stream comprising bimodal audio-visual stimuli (auditory and visual components that occur spatiotemporally consistently or inconsistently) was randomly presented to the left or to the right of the central fixation point. The visual stimulus was a white ring (an outer diameter of 7 cm and an inner diameter of 6.0 cm with a subtending visual angle of ∼6°) on a black background. The auditory stimulus was a 2000 Hz sinusoidal tone, with a linear rise and fall time of 5 ms and an amplitude of 65 dB. Auditory stimuli were presented in either the left or the right ear through an earphone (CX-300, Sennheiser, Japan). The duration of each stimulus was 15 ms. The ISI was 1500 ms. The stimuli had stimulus onset asynchronies (SOAs) between the auditory and visual stimuli ranging from −300 (negative, auditory stimulus leading) to +300 ms (positive, auditory stimulus lagging) at 20 ms intervals. The SOA was reported as 0 ms when the auditory and visual stimuli were presented simultaneously.

**FIGURE 1:**
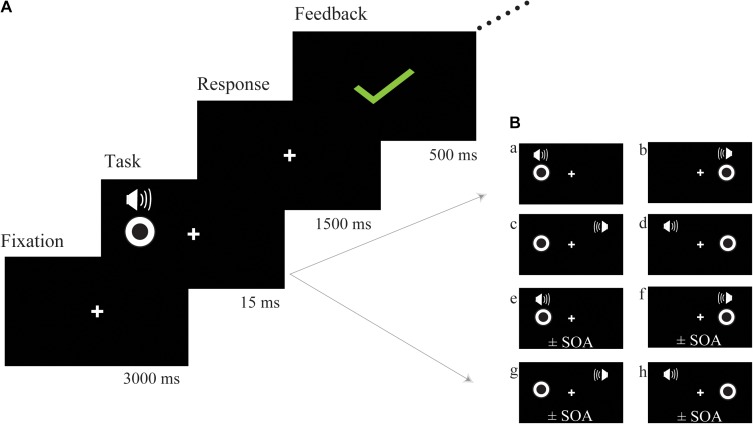
Schematic description of audio-visual training. **(A)** The trial structure of the audio-visual discrimination task. **(B)** Each subtype of audio-visual stimuli. The visual and auditory stimuli were simultaneously presented in the same hemispace (a) left (b) right; the visual and auditory stimuli were simultaneously presented in a different hemispace (c,d); the visual and auditory stimuli were asynchronously presented on the same side (e) left (f) right; the visual and auditory stimuli were asynchronously presented in a different hemispace (g,h); SOA, stimulus onset asynchrony.

The bimodal audio-visual stimulus consisted of the simultaneous or asynchronous presentation of visual and auditory stimuli in the same or in a different hemispace (see Figure 1B for a detailed description of each subtype of audio-visual stimuli). Visual and auditory stimuli were simultaneously presented in the same hemispace (left or right) or in a different hemispace (e.g., visual stimulus was presented on the left but auditory stimulus was presented on the right), and the visual and auditory stimuli were asynchronously presented in the same (left or right) or in a different hemispace. To control for response bias, we did not equally distribute each subtype of audio-visual stimuli; the ratio between consistent spatiotemporal conditions (Figure 1B, a,b) and the remaining 6 inconsistent spatiotemporal conditions was 1:6 (Figure 1B, c–h). Participants were instructed to respond to consistent spatiotemporal conditions by pressing the left button and to inconsistent spatiotemporal conditions by pressing the right button as quickly and accurately as possible. Following a response, feedback was presented with either a green checkmark or red fork corresponding to the correctness of the response (Figure 1A). Feedback was presented in the center of the screen for 500 ms. Sixteen blocks (SOA = ± 300 ms, ±280 ms, etc.) were prepared for audio-visual training, which began at ±300 ms, and each block lasted for approximately 5 min. If the discrimination accuracy of the participants exceeded 60%, the next shorter SOA block was performed. Training sessions were implemented 4 days a week and lasted from 10 to 20 min each day according to the condition of each participant, and continuous audio-visual training lasted 4 weeks. The progress of each participant was different; thus, comparing reaction times or accuracy obtained during audio-visual training was difficult. However, an audio-visual perceptual test was administered before and after training. The stimulus used in the audio-visual perceptual test was the same as the stimulation used in the training session but contained some different SOAs (±300 ms, ±250 ms, ±200 ms, ±150 ms, ±100 ms, ±50 ms, 0 ms) in one block to avoid the practice effect from the training session.

### EEG Recording and Event-Related Potential (ERP) Analysis

The control group did not perform any control task in between the two EEG recordings. The training groups may have become more attached to the study personnel and because of that be more motivated to perform well in the final oddball task. In order to avoid this effect, the members of the training groups were asked to just do training tasks in the laboratory. They did not engage in any additional communication with the study personnel. For the training and control groups, the time spent between recordings was the same. An EEG system (BrainAmp MR plus, Gilching, Germany) was used to record EEG signals through 32 electrodes mounted on an electrode cap (Easy-cap, Herrsching Breitbrunn, Germany). All signals were referenced to FCz. Horizontal eye movements were measured by deriving the electrooculogram (EOG) from one electrode placed at the outer canthi of the left eye. Vertical eye movements and eye blinks were detected by deriving an EOG from an electrode placed approximately one centimeter below the participant’s left eye. The impedance was maintained below 5 kΩ. All electrodes were offline rereferenced to the average of both mastoids. The EEG and EOG signals were amplified and bandpass filtered with an analog filter of 0.01–100 Hz. Raw signals were digitized using a sample frequency of 500 Hz with a 60 Hz notch filter.

ERP data were analyzed using Brain Vision Analyzer software (Version 2.0, Brain Products GmbH, Munich, Germany), and ERP data were averaged separately for each stimulus type off-line. EEG and EOG signals were divided into epochs from 100 ms before the stimulus onset to 600 ms after onset, and baseline corrections were made from −100 to 0 ms relative to stimulus onset. Epochs contaminated with large artifacts were identified using the following standard: vertical EOG amplitudes exceeding ±120 μV, horizontal EOG amplitudes exceeding ±35 μV, and a voltage exceeding ±100 μV at any electrode location relative to baseline. These trials were subject to automatic rejection from the analysis. Then, the remaining trials were averaged separately for each participant, each session and each stimulus type following digital filtering using a bandpass filter of 0.01–30 Hz. The peak amplitude was measured relative to the prestimulus baseline. The P300 component was defined as the largest positive deflection within 300 and 600 ms. Peak latency was defined as the time from stimulus onset to the peak of each scalp component. The grand-averaged data were obtained across all participants for two auditory stimulus types (1000 and 2000 Hz). A total of 160 trials to non-target tones and 40 trials to target tones were obtained for offline averaging of the signal.

The P300 amplitudes were analyzed with 2 Time (pretest and posttest) ^∗^ 3 Electrode (Fz, Cz, and Pz) using analysis of variance (ANOVA), and the alpha level was set at *P* < 0.05. The Greenhouse-Geisser Epsilon correction was applied to adjust the degrees of freedom of the F ratios as necessary. All statistical analyses were carried out using SPSS version 16.0 software (SPSS, Tokyo, Japan).

## Results

All participants had normal or corrected-to-normal vision and normal hearing, and none used a hearing aid. When the older adult participants performed an auditory perception task in an auditory oddball paradigm, the correct rate exceeded 90%. The cognitive status of all participants was in the normal range regardless of older adults’ pretest (training group: MMSE = 28.9 ± 0.9, MoCA = 27.6 ± 1.0; control group: MMSE = 28.5 ± 0.8, MoCA = 27.3 ± 0.9) or posttest (training group: MMSE = 29.0 ± 0.8, MoCA = 27.5 ± 0.9; control group: MMSE = 28.7 ± 1.0, MoCA = 27.4 ± 0.8) scores or young adults’ pretest (training group: MMSE = 29.5 ± 0.6, MoCA = 28.2 ± 0.7; control group: MMSE = 29.7 ± 0.5, MoCA = 28.3 ± 0.7) or posttest (training group: MMSE = 29.7 ± 0.4, MoCA = 28.3 ± 1.0; control group: MMSE = 29.8 ± 0.4, MoCA = 28.5 ± 0.7) scores. Analyses revealed that older adults’ MMSE [*F*(1,48) = 23.795, *P* < 0.001, $\eta_{P}^{2}$ = 0.331] and MoCA [*F*(1,48) = 15.669, *P* < 0.001, $\eta_{P}^{2}$ = 0.246] scores were significantly lower than those of young adults. There were no significant differences in other factors, suggesting that audio-visual perceptual training does not directly affect MMSE and MoCA scores.

The accuracy of each stimulus type is presented in Table 1 for both young and older adults in the audio-visual perceptual test. We performed 2 ^∗^ 2 ^∗^ 2 ^∗^ 13 ANOVA with Time (pretest, posttest) and Modality (SOAs) as within-subject factors, Age (young adults, older adults) and Group (training, control) as a between subjects factor. A main effect of Time [*F*(1,48) = 4.843, *P* = 0.033, $\eta_{P}^{2}$ = 0.092], Modality [*F*(12,576) = 117.554, *P* < 0.001, $\eta_{P}^{2}$ = 0.710], and Age [*F*(1,48) = 64.805, *P* < 0.001, $\eta_{p}^{2}$ = 0.574] was found, but there was no significant main effect of Group [*F*(1,48) = 2.176, *P* = 0.147, $\eta_{P}^{2}$ = 0.043]. Moreover, there was a significant interaction between Time and Group [*F*(1,48) = 7.564, *P* = 0.008, $\eta_{P}^{2}$ = 0.136], and between Age and Modality [*F*(12,576) = 21.007, *P* < 0.001, $\eta_{P}^{2}$ = 0.304]. Interactions between other factors were non-significant. Pairwise comparisons revealed that accuracy was significantly higher after training (*P* = 0.001) than before training. However, there was no significant difference (*P* = 0.699) in the accuracy between the pretest and posttest for the control group. These behavior results indicated that the accuracy of audio-visual perception was improved after training for both young and older adults.

**Table 1:** Mean accuracy in the audio-visual perceptual test for young and older adults.

Stimulus types	Young adults				Older adults			
	Training		Control		Training		Control	
	Pretest	Posttest	Pretest	Posttest	Pretest	Posttest	Pretest	Posttest
AV	85.3 (2.8)	87.5 (1.4)	78.3 (6.4)	83.1 (4.8)	67.5 (5.1)	78.3 (2.9)	71.1 (3.3)	67.2 (5.2)
A300V	76.8 (4.5)	87.3 (3.3)	68.0 (7.9)	70.4 (9.1)	24.3 (6.1)	55.0 (6.2)	31.1 (6.8)	30.6 (6.6)
A250V	68.4 (5.3)	85.3 (4.5)	64.9 (7.8)	67.1 (9.4)	23.7 (6.3)	39.6 (7.9)	30.2 (4.7)	29.5 (5.8)
A200V	64.4 (6.3)	86.1 (3.3)	60.8 (7.2)	65.8 (8.7)	20.1 (5.3)	35.8 (6.8)	25.1 (3.6)	26.5 (4.8)
A150V	59.5 (5.7)	77.4 (4.9)	54.2 (6.5)	62.9 (9.2)	19.5 (4.8)	29.5 (7.3)	21.6 (4.1)	24.7 (4.1)
A100V	37.2 (6.2)	68.9 (4.9)	44.1 (6.1)	51.4 (7.1)	19.4 (4.6)	21.9 (5.2)	19.5 (4.3)	20.5 (4.6)
A50V	21.6 (3.6)	32.4 (7.5)	28.5 (5.8)	22.5 (5.6)	17.5 (4.5)	15.0 (3.8)	20.2 (4.2)	19.2 (4.3)
V50A	14.9 (4.1)	14.5 (2.8)	22.0 (7.4)	10.1 (2.9)	14.2 (3.9)	13.1 (3.1)	12.5 (2.9)	15.9 (3.7)
V100A	17.2 (4.3)	21.8 (4.3)	24.8 (7.8)	14.0 (3.8)	12.4 (3.4)	16.3 (4.6)	16.1 (4.1)	13.7 (3.3)
V150A	19.2 (3.8)	31.5 (5.8)	30.2 (7.1)	18.2 (5.2)	14.8 (3.6)	15.4 (3.5)	17.4 (3.4)	16.3 (3.4)
V200A	26.8 (4.6)	40.8 (9.1)	41.7 (8.5)	26.5 (7.4)	13.4 (3.9)	21.8 (4.9)	17.2 (3.8)	15.4 (3.7)
V250A	33.3 (6.4)	46.1 (8.6)	36.3 (7.1)	33.4 (8.3)	16.4 (4.1)	24.9 (4.9)	18.8 (3.8)	17.0 (3.9)
V300A	42.5 (7.4)	54.6 (8.9)	43.2 (6.4)	35.0 (8.2)	19.1 (4.9)	45.6 (5.4)	20.5 (5.0)	21.3 (4.8)

*Standard errors of the mean are given in parentheses.*

**FIGURE 2:**
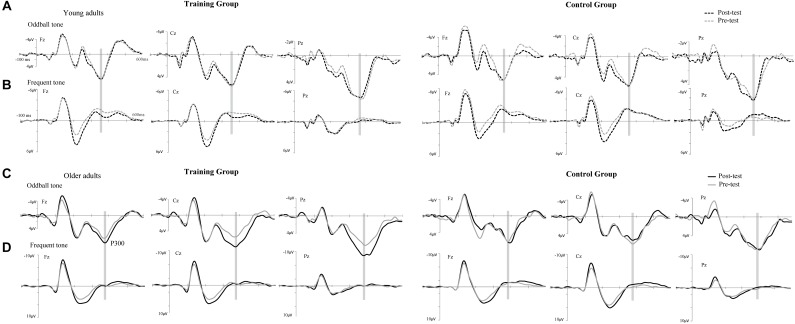
Grand-average event-related potentials (ERPs) averaged across electrodes Fz, Cz, and Pz elicited by an auditory oddball paradigm in the training (left side) and control groups (right side) for both young **(A,B)** and older adults **(C,D)**. Mean P300 amplitudes elicited by target stimuli in the pretest (gray line) and posttest (black line), electrodes Fz, Cz, and Pz in a time window between 360 and 370 ms for young adults and between 380 and 390 ms for older adults (gray shadow).

**FIGURE 3:**
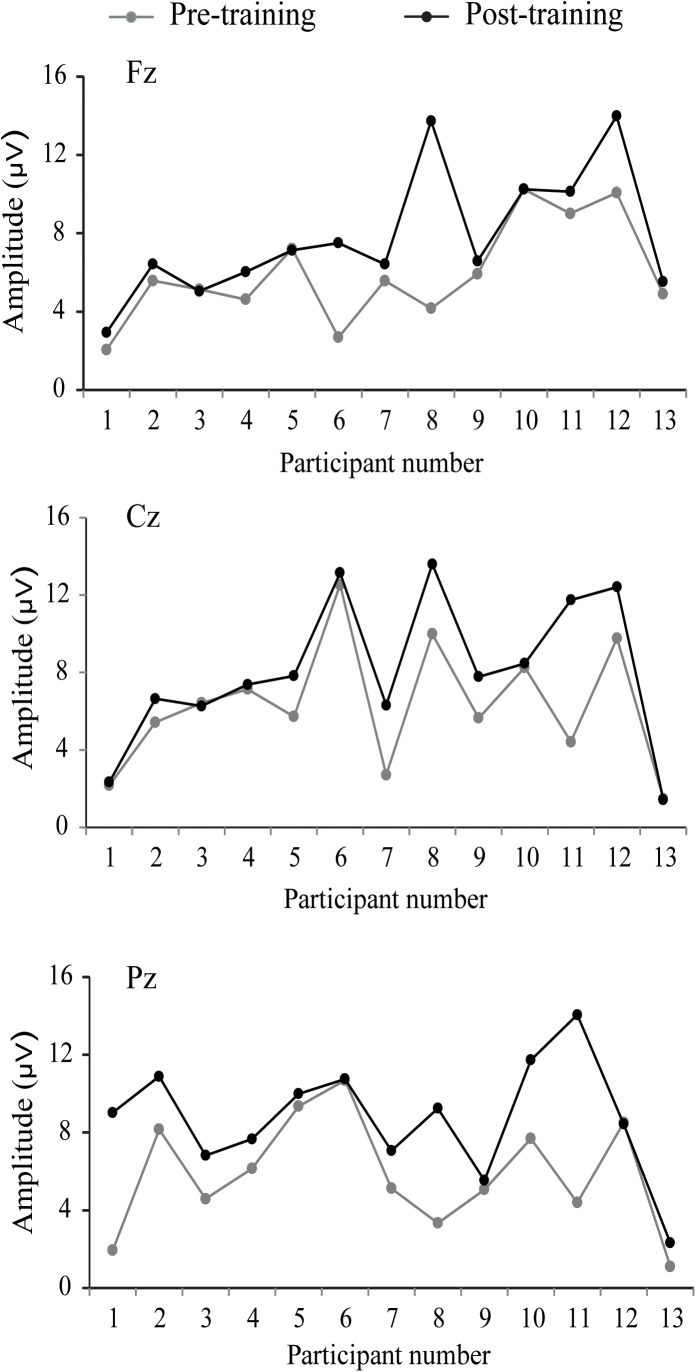
Individual P300 amplitudes and changes before and after training for older adults.

The P300 components elicited by an auditory oddball paradigm in the pre- and posttest sessions are presented in Figure 2. To identify any differences in the P300 amplitude between the pretest session and posttest session in both young and older adults, we performed 2^∗^ 2^∗^ 2 ^∗^ 3 ANOVA with Time (pretest, posttest) and Electrode (Fz, Cz, and Pz) as within-subjects factors and Age (young adults, older adults) and Group (training, control) as a between subjects factor. A main effect of Time [*F*(1,48) = 4.740, *P* = 0.034, $\eta_{P}^{2}$ = 0.09] was detected, but there was no significant main effect of Age [*F*(1,48) = 2.264, *P* = 0.139, $\eta_{P}^{2}$ = 0.045], Group [*F*(1,48) = 0.852, *P* = 0.361, $\eta_{P}^{2}$ = 0. 017], or Electrode [*F*(2,96) = 1.012, *P* = 0.365, $\eta_{P}^{2}$ = 0.021]. Moreover, there was a significant interaction between Time and Age [*F*(1,48) = 4.815, *P* = 0.033, $\eta_{P}^{2}$ = 0.091], between Time and Group [*F*(1,48) = 4.441, *P* = 0.040, $\eta_{P}^{2}$ = 0.085], and among the three factors [*F*(1,48) = 5.007, *P* = 0.030, $\eta_{P}^{2}$ = 0.094]. Interactions in between other factors were non-significant. For young participants, the comparison across time points revealed no significant improvement between the pretest and posttest for both the control (pretest: *M* = 5.628 μV, posttest: *M* = 5.660 μV, 95% CI [−1.153, 1.090], *P* = 0.955) and training group (pretest: *M* = 5.731 μV, posttest: *M* = 5.690 μV, 95% CI [−1.080, 1.163], *P* = 0.942). For older adults, there was also no significant difference between pre-test (*M* = 6.026 μV) and post-test (*M* = 6.033 μV) in the control group (95% CI [−1.129, 1.114], *P* = 0.990). Nevertheless, significantly different P300 amplitudes (95% CI [−3.553, −1.310], *P* < 0.001) were found between the pretest (*M* = 5.760 μV) and posttest (*M* = 8.191 μV) in the training group (Figure 3). These results indicated that the P300 amplitude was enhanced after 1 month of audio-visual perception training in older adults.

Additionally, ANOVA of P300 latency revealed a main effect of Age [*F*(1,48) = 42.208, *P* < 0.001, $\eta_{P}^{2}$ = 0.468], showing an earlier peak in young adults than in older adults (Table 2). No other main effects (all *P*-values >0.05) or interactions (all *P*-values >0.05) were significant. Therefore, no significant differences were found for P300 latency between the post- and pretraining sessions for both young and older adults.

**Table 2:** The mean amplitudes and latencies of P300 for both young and older adults.

	Young adults						Older adults					
	Training			Control			Training			Control		
	Fz	Cz	Pz	Fz	Cz	Pz	Fz	Cz	Pz	Fz	Cz	Pz
**Amplitude (μV)**												
Pretest	5.46 (0.98)	5.58 (1.10)	6.28 (1.36)	5.47 (0.70)	5.37 (0.81)	6.04 (0.84)	5.93 (0.71)	5.39 (1.36)	5.94 (0.76)	5.92 (0.53)	6.01 (0.62)	6.14 (0.74)
Posttest	5.59 (0.68)	5.46 (0.66)	6.08 (1.09)	5.26 (0.67)	5.53 (0.71)	6.19 (0.79)	7.83 (0.91)	8.11 (1.05)	8.64 (0.89)	6.11 (0.55)	5.69 (0.57)	6.29 (0.64)
**Latency (ms)**												
Pretest	367 (2.92)	368 (2.63)	369 (2.37)	365 (3.18)	367 (3.91)	368 (4.23)	385 (7.74)	370 (6.30)	384 (11.02)	387 (5.38)	376 (4.89)	384 (4.87)
Posttest	368 (2.88)	367 (3.15)	369 (2.74)	370 (4.05)	364 (4.16)	364 (4.67)	377 (4.35)	372 (7.99)	385 (9.43)	391 (5.44)	382 (5.86)	388 (6.59)

*Standard errors of the mean are given in parentheses.*

## Discussion

This study investigated the influences of cross-modal audio-visual spatiotemporal perceptual training on the cognitive ability of young adults and healthy older adults. Participants were trained on spatiotemporal discrimination ability, an important cognitive ability. Before and after spatiotemporal discrimination training, cognitive ability was assessed using the P300 ERP component which was elicited by an auditory oddball task. P300 latency elicited by the auditory oddball paradigm was earlier in young adults than in older adults. Our results were in agreement with the findings obtained in previous studies, in which showed the latencies of P300 were significantly higher in older adults than in young adults (Amenedo and Díaz, 1998). However, no significant differences were found between the post and pretraining sessions for young adults. A possible reason why young adults did not improve in this task is that they already performed very well on the pretraining evaluation and during the training task. By contrast, in older adults, the P300 amplitudes in the posttraining sessions were significantly greater than those in the pretraining sessions. These results indicate that audio-visual perceptual training enhanced the P300 component in healthy older adults. Some studies evaluating the effects of aging have shown greater response time facilitation in cross-modal audio-visual stimuli than in unimodal stimuli in both young and older adults (Laurienti et al., 2006; Peiffer et al., 2007). The results of the aforementioned studies showed that audio-visual integration in older adults was greater than that in young adults, indicating that older adults could benefit more from combining information from visual and auditory modalities. These results also indicated that multiple sensory channels used an effective compensatory strategy to overcome the unisensory deficits associated with aging, and further suggested that the enhanced audio-visual integration in older adults might be due to changes in multisensory processing. Thus, audio-visual perceptual training may be more beneficial than unimodal visual or auditory training for improving the cognitive ability of older adults.

For older adults, effective training of perceptual abilities can potentially impact a number of cognitive functions (Lindenberger and Ghisletta, 2009). Setti et al. (2014) found that audio-visual temporal order judgment training affected multisensory integration, indicating that plasticity is preserved in audio-visual perceptual discrimination abilities in older adults. Their results further suggested that perception training can induce a far-transfer effect in other untrained perceptual processes that are not directly trained (Setti et al., 2014). P300, a thoroughly examined late ERP component, is considered to reflect attentional functions, including attentional resource allocation and attentional reorientation (Polich, 2007). Moreover, compared with controls, adult patients with attention deficit hyperactivity disorder have a significantly reduced P300 amplitude across multiple studies (Szuromi et al., 2011). In the present study, cross-modal temporal and spatial discrimination training, which requires a rapid shift in attention between the two modalities, may have improved the ability of older adults to attend to a stimulus. Thus, cross-modal audio-visual training may raise attention, contributing to the enhancement of P300 amplitude. Another possible explanation for this improvement is the potential association with long-term memory consolidation (McGaugh, 2000). Some ERP results clearly show that the P300 amplitude of the high-ability (fluid intelligence) group was relatively larger than that of the low-ability group, suggesting that P300 amplitude is associated with learning and memory (Amin et al., 2015). Therefore, continuous training for 1 month may play an important role in strengthening the original training effects for older adults.

In summary, the current study shows that P300 amplitude in older adults can be enhanced by cross-modal audio-visual perception training, suggesting that this training may be effective in improving the cognitive ability of older adults. Another important point is that this training is simple and easy to implement for older adults, providing a theoretical basis for the subsequent development of training equipment specifically for older adults. However, one limitation of this study is the small sample size for participants. Therefore, further studies are needed to confirm it in details, as well as elucidate the cognitive processes affected by this training and whether it is beneficial to everyday life.

## Author Contributions

WY wrote the paper. AG analyzed and interpreted the data. YL and JQ performed the experiments. YR and WY conceived and designed the experiments. WY, YR, SY, SL, and JC revised the paper and approved the final version of the manuscript.

## Conflict of Interest Statement

The authors declare that the research was conducted in the absence of any commercial or financial relationships that could be construed as a potential conflict of interest.
